# Colloidal and membrane stabilization of nanoproliposomes coated with chitosan-alginate polymers: Thermal properties, gastrointestinal release and reconstitutability

**DOI:** 10.1016/j.crfs.2026.101315

**Published:** 2026-01-21

**Authors:** Zahra Akbarbaglu, Fardin Tamjidi, Khashayar Sarabandi, Seid Mahdi Jafari

**Affiliations:** a Department of Food Materials & Process Design Engineering, Gorgan University of Agricultural Sciences and Natural Resources, Gorgan, Iran; b Department of Food Science & Engineering, Faculty of Agriculture, University of Kurdistan, Sanandaj, Iran; c Department of Food Chemistry, Research Institute of Food Science and Technology (RIFST), Mashhad, Iran; d Halal Research Center of IRI, Iran Food and Drug Administration, Ministry of Health and Medical Education, Tehran, Iran

**Keywords:** Phycocyanin, Polymer complex, Biological stability, Controlled release

## Abstract

Instability, leakage and unwanted release of bioactive compounds loaded into nanoliposomes (NLs) are among the key challenges during storage, formulation, or various tensions (mechanical, thermal, shear, etc.). In this study, lipid membrane stabilization of phycocyanin (PC)-containing NLs was performed through chitosan-alginate (NAs) bilayer coating. Then, the reconstitution capability and retention of the biological properties of nanoproliposomes (NPLs) was investigated. The polymer complex led to improved nanovesicle properties (216 nm, −33.6 mV), encapsulation efficiency (EE 78.2 %), physical stability, EE-retention, bioactive contents (PC and TPC), biological activities (DPPH∗/ABTS∗ scavenging), and morphology (SEM), during different tensions (thermal, light and freezing) as well as controlled release (under gastrointestinal conditions). The properties of NPLs (production yield (48–62 %), flowability (relative cohesion and compressibility), functional indices (solubility ∼93 %), hygroscopicity, retention of bioactives (∼86–96 %), histogram and color indices) were affected by the composition and type of coating. Chemical (FTIR) and thermal (DSC) evaluations indicated electrostatic adsorption of biopolymers and increased rigidity of the coated membrane. The transformation of brittle structures (FD-B-NLs) into compact and rough (FD-NAs) was confirmed by SEM images. NPLs showed particles (∼430–590 nm) with wrinkled, indented structures and intact walls. The polymer bilayer coating resulted in maintaining membrane strength, structural stability, reduced unwanted leakage (75–82 % of the initial EE), and particle morphological changes during shear/dehydration stresses after reconstitution. The findings of this study can be used to design powder formulations containing stable pharmaceutical and food nanocarriers.

## Highlights

• **The production of nano-proliposomes coated with chitosan-alginate complex was investigated.**
• **Electrostatic adsorption and lipid membrane stabilization were confirmed by FTIR and DSC.**
• **The biopolymer complex resulted in biological-structural stability, and controlled release of particles.**
• **The properties of proliposomes were influenced by the composition of nanoparticles and polymers.**
• **DSL, SEM, AFM, and TEM analyses confirm structural stability of coated NLs post-reconstitution and under tensions.**

## Graphical abstract

**Figure:**
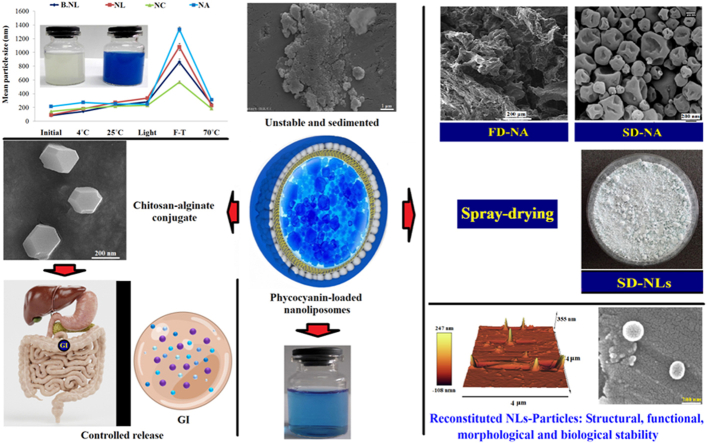

## 1 Introduction

In recent years, lipid nanocarriers have gained a special place in the food and pharmaceutical industries. In particular, new achievements have been made in the design and production of drug-loaded lipid delivery systems (nanodrugs) with the aim of treating diseases such as cancer, cardiovascular, neurological, diabetes, and inflammation (Choudhary et al., 2023; Laein et al., 2024). The most important applications of these systems include: (i) masking undesirable aromas and flavors (for food ingredients); (ii) physicochemical and thermal stabilization; (iii) bio-stability; (iv) increasing solubility and bioavailability; (v) modifying biodistribution; (vi) limited controlled and targeted delivery (Bai et al., 2011; Karim et al., 2020; Sepúlveda et al., 2021) These benefits have led to an increase in the value of the global nanomedicine market from approximately $135 billion (in 2015) to over $350 billion (in 2025)(Shah et al., 2020). Among lipid nanocarriers, nanoliposomes (NLs) are of great importance due to their structural features and phospholipid composition (biodegradable, biocompatible nature, and similarity to cell membrane lipids), and the ability to simultaneously load lipophilic and hydrophilic compounds (Marín et al., 2018).

Despite numerous advantages, challenges such as oxidation of phosphatidylcholine, microbial spoilage, physical instability, electrostatic and membrane fragility (e.g., phase separation, creaming, Ostwald ripening, agglomeration and coalescence), and leakage/fusion and unwanted release of loaded compounds have led to technological limitations of NLs (Laein et al., 2024; Moosavi-Nasab et al., 2023). In particular, particle size, surface charge, chemical stability, and loading capacity (LC) are key indices for maintaining the performance, controlled delivery, and bioavailability of bioactive compounds (Shah et al., 2020). In this regard, numerous efforts have been made regarding structural stabilization, formulation modification, and changes in phospholipid composition and vesicle membrane. These investigations include liposomes modified with decanoic acid and stearic acid (Huang et al., 2024), partial replacement of phosphatidylcholine with hydrogenated phosphatidylcholine (Zhu et al., 2024), addition of trehalose (Sepúlveda et al., 2021), and various stabilizers (cholesterol or glycerol (Chotphruethipong et al., 2020). On the other hand, coating NLs with biopolymers (such as carbohydrates and proteins) has been associated with beneficial results due to their biocompatible nature, biodegradability, low cost, and natural availability (Akbarbaglu et al., 2024). In this regard, the effects of coating NLs with polymers such as chitosan (CH)(Ma et al., 2022), pectin (Haghighi et al., 2018; Karim et al., 2020; Shishir et al., 2019), sodium alginate (SA)(W. Liu et al., 2017), SA and genipin (Ghaleshahi and Rajabzadeh, 2020), gelatin (Hosseini et al., 2021), and carrageenan, trehalose, and pectin (Cong et al., 2023) on the stabilization of a variety of bioactives (e.g., oyster protein hydrolysates, phloridzin, pelargonidin-3-O-glucoside, neohesperidin, vitamin C, ω-3 PUFAs, and quercetin) were investigated.

Another way to increase long-term stability (physical, chemical, and microbial), reduce food/drug interaction (compared to the liquid state), minimize unwanted release of loaded compounds during long-term storage, and facilitate storage, packaging, and transportation is to convert liquid NLs formulations into solid (powder) form using freeze-drying (FD) and spray-drying (SD)(Laein et al., 2024; Taşdemir and Gölge, 2024). Therefore, the production of vitamin D_3_-loaded proliposomes has been carried out using the micronized sucrose (MSC) coating technique (Chaves and Pinho, 2020), drying liposomal suspensions in alginate matrix (Gómez-Estaca et al., 2021), liposomes with encapsulated black carrot extract (Guldiken et al., 2019), and carboxymethyl CH-coated proliposomes containing coix seed oil (Bai et al., 2011).

Legal restrictions or some potential risks associated with artificial colors from regulatory and supervisory bodies (such as FAO, WHO, and EFSA) have led to increased attention to natural alternatives. This trend has led to an increase in the share of natural alternatives (∼25 %) in the total value of the global food colouring market (∼3.75 billion USD) (Rodríguez-Mena et al., 2022). Among a variety of bioactives, phycocyanin (PC) from blue-green algae or cyanobacteria can be considered as a peptide-based pigment with positive biological and health-promoting effects in food and pharmaceutical purposes. Hence, its global market value (around US$245.5 million by 2027) has increased significantly (Ashaolu et al., 2021). However, the peptide nature and sensitivity of PC to environmental factors (heat, light, acid, high pressure, heavy metal cations, and denaturing agents) or digestive conditions have caused the loss of its functional, biological, bioavailability, and color properties (Shafieiyoun et al., 2024; Wijesekara and Xu, 2024).

Considering the bioactive properties, the necessity of PC encapsulation and the design of a stable delivery system, as well as investigating the effects of bilayer membrane coating on maintaining the physical properties, stability, morphology, and reconstitution capacity of the particles, the objectives of this study included the following: Investigating the effects of different concentrations of alginate coating on 1) physical properties, zeta potential (ZP), EE in PC-loaded NLs and determining the optimal sample; 2) Evaluation of physical stability, EE retention and morphological structures (SEM) in NLs, nanochitosome (NCs) and bilayer-coated (NAs) under thermal, light and freezing stresses; 3) Investigating the stability of bioactives (PC, TPC) and biological activities (scavenging of DPPH and ABTS radicals) under the influence of coating (monolayer and bilayer) and different stresses; 4) Evaluation of the release of PC loaded into NLs/NCs/NAs under digestive conditions (stomach and intestine); 5) Investigation of the effects of NLs coating on production yield (PY), physical properties, flowability, hygroscopicity, particle size, and retention (%) of PC, TPC, and scavenging of DPPH and ABTS radicals in spray-dried nanoparticles; 6) Evaluation of histograms and color indices, thermal (DSC), chemical (FTIR), and morphological (SEM) properties of particles; 7) Comparison and evaluation of physical properties (size and PDI), ZP, EE in freeze-dried and spray-dried nanoparticles after reconstitution; 8) Investigation of morphological (SEM), topographic (AFM), and structural (TEM) properties in reconstituted NLs and NAs powders.

## 2 Materials and methods

PC powder (extracted from *Arthrospira platensis*) was provided from Arian Gostar Co (Tehran, Iran). Sodium alginate (SA: A0682, low viscosity, 12 KDa), CH (448869, viscosity <50 mPa s, 50 KDa), Cholesterol, DPPH (1,1-Diphenyl-2-picrylhydrazyl) were provided from Sigma–Aldrich Co. (St. Louis, MO, USA). PHOSPHOLIPON® 85G (Lipoid, Germany) was prepared. Folin-Ciocalteu reagent, Gallic acid (GA), sodium carbonate, and other chemicals were purchased from Merck (Darmstadt, Germany).

### 2.1 PC-loaded nanoliposomes

NLs were prepared using a modified method based on Akbarbaglu et al. (2024). Specifically, 0.18 g phosphatidylcholine (wall material), 0.02 g cholesterol (which enhanced membrane stiffness), and 0.05 g Tween-80 (emulsifier) were dissolved in 20 mL absolute ethanol over 20 min. This solution was transferred to a 250 mL round-bottom flask, and the solvent was evaporated using a rotary evaporator at 60 °C and 70 rpm to form a thin lipid film. The film was hydrated with 20 mL phosphate-buffered saline (PBS, pH 7.4, 0.05 mol/L) containing PC (6 mg/mL). The mixture was vortexed for 2 min and was stirred in a rotary evaporator at 60 °C. Finally, the suspension placed in a beaker containing cold water (4 °C) was sonicated using an ultrasound probe (UP200H, Hielscher, Germany) at 20 kHz for 15 min with 2-s pulses to produce NLs. The final liposomal system consisted of phosphatidylcholine (9 mg/mL), cholesterol (1 mg/mL), and Tween 80 (2.5 mg/mL).

### 2.2 Nanochitosomes preparation

The CH-coated NLs (NCs) were prepared based on the method of Shishir et al. (2021) with minor alterations. CH was dissolved in 1 % acetic acid (v/v) at 0.4 % w/v for 12 h. Then, the same volume of each CH solution was added dropwise to the NL solution while stirring at 300 rpm. The coated samples were preserved at 4 °C until testing.

### 2.3 Double-layer coated NLs (NAs)

To produce NCs with alginate (NAs), the method of Liu et al. (2017) was used with some modifications. Following preliminary iterative testing and noting system destabilization at alginate concentrations exceeding 0.4 % w/v, an operational alginate concentration window of 0.10–0.40 % w/v was selected. Alginate was dissolved (0.1, 0.2, 0.3, and 0.4 % w/v) in distilled water (pH 5.5) and was stirred for 12 h. The resulting solution was centrifuged (3000×*g*, 15 min). Then, an equal volume of CH-NL solution was added dropwise to the alginate solution while stirring.

### 2.4 Nanovesicles characterisation

#### 2.4.1 Mean particle size, PDI, and zeta potential

The size, PDI, and ZP of the NLs/NCs/NAs were measured by using a dynamic light scattering (DLS) system (Nano-Sizer 3000, Malvern Instruments, Malvern, UK) at 25 °C and an angle of 90°, after individual samples were diluted with distilled water to a dilution factor of 100.

#### 2.4.2 PC-Encapsulation efficiency (EE)

The EE of PC was determined using the method of Gharehbeglou et al. (2024) with some modifications. Briefly, 2 mL of uncoated NLs and coated NLs (NCs/NAs) were transferred to a 30 KDa cut-off Amicon filter and then centrifuged at 3000 rpm for 10 min. Finally, the EE was determined by measuring the concentration of PC loaded into the NLs/NCs/NAs as a percentage of the total PC. The calculation formulas were as follows.

(1) EE (%) = (Encapsulated PC (mg)/Total PC (mg)) × 100

#### 2.4.3 Bioactivity and antioxidant characterization

##### 2.4.3.1 PC concentration

Determination of PC concentration was performed as reported by İlter et al. (2018) with slight modification. The absorbance of a 2 mL sample was recorded with a spectrophotometer (PJ Instruments, model T80, England) at 620 and 652 nm, and the concentration of PC (w/v) was calculated using the following equation:

(2) $$\text{PC}\;\left(\text{mg}/\text{mL}\right)=\frac{A_{620-}0.475\times A_{652}}{5.34}$$

##### 2.4.3.2 Total phenolic content (TPC)

TPC was measured based on the method of Guldiken et al. (2019) with slight alterations. In brief, 50 μL of each sample was mixed with 2.3 mL distilled water and 200 μL Folin–Ciocalteu reagent. After 5 min, 600 μL of Na_2_CO_3_ solution (20 %) was added and the mixture was incubated at room temperature for 30 min. Subsequently, the absorbance was recorded at 760 nm (PJ Instruments, model T80, England). Gallic acid was used as the calibration standard.

##### 2.4.3.3 DPPH∗ scavenging

For the DPPH assay, 0.5 mL of extract solution was mixed with 1 mL of 0.2 mM DPPH solution, and the resulting solution was kept in the dark for 30 min. Then the mixture was centrifuged (4000×*g*, 10 min) and the supernatant absorbance was recorded at 517 nm (Ma et al., 2022). The DPPH radical scavenging (RSA) was calculated using the following formula:

(3) RSA (%) = [1- (sample _Abs_/blank _Abs_)] × 100

##### 2.4.3.4 ABTS∗ scavenging

The ABTS solution was prepared from ABTS (7 mM) and potassium persulfate (2.45 mM). After storage in the dark for 12 h, the mixture was diluted with 0.2 M PBS (pH 7.4) to an absorbance of 0.70 at 734 nm. Then 50 μL of PC or nano (NLs/NCs/NAs) solution was added to 2 mL of the ABTS solution, vortexed for 30 s, and kept in the dark for 6 min. After that, the absorbance was recorded at 734 nm and ABTS-RSA was determined using equation (3) (Gharehbeglou et al., 2024).

#### 2.4.4 Physical, E.E and biological stability

In this study, the effects of different tensions (refrigeration at 4 °C, ambient temperature at 25 °C, heating at 70 °C, light exposure, and freeze–thaw cycles) on physical stability (particle size), EE retention, morphological changes (SEM and TEM), and biological activities (PC, TPC, DPPH, and ABTS-RSA) were investigated.

##### 2.4.4.1 Thermal and photo-stability

Samples (2 mL each) were stored for 28 days at 4 °C in the refrigerator, at 25 °C in the dark, and under white fluorescent light (100 Lux, 40 cm distance) at 25 °C. Thermal stability was further evaluated by heating the samples (in an oven) at 70 °C for 60 min (Shishir et al., 2021).

##### 2.4.4.2 Freeze-thaw stability

Samples were frozen at −18 °C for 4 weeks, then thawed at room temperature (25 °C) and mixed by vortexing for 1 min. After this process, changes in mean particle size, EE, PC content, TPC, and free radical scavenging (DPPH and ABTS assays) were measured using established methods (2.4.3.1–2.4.3.4) (Akbarbaglu et al., 2024).

#### 2.4.5 Evaluation of *in vitro* release

The study assessed EE stability of NLs, NCs and NAs under simulated digestive conditions to evaluate the release behavior of encapsulated peptides. Simulated gastric fluid (SGF) was prepared with pepsin (100 mg), hydrochloric acid (0.35 mL), and sodium chloride (100 mg) at pH 1.2, while simulated intestinal fluid (SIF) contained monobasic potassium phosphate (340 mg), sodium hydroxide (3.85 mL of 0.2 M), and pancreatin (500 mg) at pH 6.8. Samples of 100 μL were diluted to 2 mL with either SGF or SIF, and then incubated at 37 °C with shaking at 300 rpm for 4 h (gastric) or 2 h (intestinal). Phycocyanin (PC) release at intervals of 0.5–1 h was calculated according to the following equation (Zhu et al., 2024):

(4) PC release (%) = (Released PC (mg)/Total PC (mg)) × 100

### 2.5 Spray-drying of nanoliposomes

For preparation of the feed, 2 g maltodextrin (MD) was dissolved in 20 mL distilled water for 1 h at 300×*g*. Afterward, 10 mL liposomal solution (B-NLs/NLs/NCs/NAs) was added to 10 mL MD solution. The feed-to-powder conversion process was performed with a lab-scale spray-dryer (Büchi B-290, Switzerland). The process parameters encompassed the inlet-air temperature (140 °C), outlet-air temperature (78 °C), feed rate (5 mL/min), drying air speed (0.54 m3/h), nozzle diameter (0.5 mm), and air pressure (5.4 bar). To evaluate the effect of coating on the structural properties (SEM) of the particles, each sample (without MD) was freeze-dried (Christ, Germany) at −20 °C under a pressure of 0.1 mbar. The SD and FD powders were selected, packed in airtight bags, and kept in a refrigerator until experiments.

#### 2.5.1 Physicochemical and biological properties of SD-samples

Production yield, physical and functional properties of powders such as moisture content (MC), water activity (aw), bulk (BD) and tapped (TD) densities, flowability (angle of repose (AR), Hausner ratio (HR), Carr index (CI)), solubility, hygroscopicity, and mean particle size were determined (Li et al., 2015). Also, PC, TPC values, and DPPH and ABTS assays were calculated.

#### 2.5.2 Color analysis

The color factors were measured by L∗, a∗, and b∗ values. Images of samples were taken by a Canon digital camera (PowerShot A3400) and color analysis was performed by means of ImageJ software (http://imagej.net). The hue angle and chroma were determined using the subsequent formula:

(5) Hue = tan^−1^ (b∗/a∗)

(6) Chroma = [(a∗)^2^+(b∗)^2^]^1/2^

### 2.6 Morphological characterization

#### 2.6.1 Scanning Electron Microscopy (SEM)

The morphological and structural characteristics of primary and stored NLs, NCs, and NAs were examined using a Hitachi SEM (model PS-230, Japan). These evaluations were conducted subsequent to gold coating and by utilizing an accelerating voltage of 25 kV (Ma et al., 2022).

#### 2.6.2 Atomic Force Microscopy (AFM)

In this study, tapping mode was employed to determine the phase contrast and topography of the samples (Eid et al., 2021). For this process, a drop of each sample (diluted 100-fold) was placed on a glass slide and dried at room temperature. Subsequently, the structure of each sample was examined using an Agilent 5420 microscope (Keysight, California, USA).

#### 2.6.3 Transmission Electron Microscopy (TEM)

The evaluation of nano-structures with TEM was conducted using the negative staining method (Pavlović et al., 2022). After dilution, NLs were combined with ammonium molybdate solution and a drop of the mixture was placed on a carbon-coated grid (mesh 200, diameter 3 mm) and allowed to settle for 5 min. After drying the sample at room temperature, the structure of the vesicles was assessed by TEM (JEM2100, JEOL, Japan).

### 2.7 Thermal properties (DSC)

Differential scanning calorimetry (DSC) was performed using a DSC instrument (DSC Q2000, Waters, Milford, MA, USA) to determine the glass transition temperature (Tg) of the microcapsules. Approximately 5 mg of powder was placed in sealed aluminum pans. The samples were heated from −20 to 240 °C at a rate of 10 °C/min in a dynamic artificial air environment (20 mL/min). Before analysis, the system was calibrated using indium as a reference standard (Bai et al., 2011).

### 2.8 Structure analysis (FTIR)

To analyze the structure by FTIR, samples including pure PC, empty (B-NL) and loaded NLs, NCs, and NAs, and SD samples were blended with potassium bromide (KBr) and then compressed into disk-shaped forms. FTIR spectroscopy of the samples was performed using an FTIR spectrophotometer (Shimadzu 8400, Japan) scanning frequencies ranging from 4000 to 400 cm^−1^ (Pavlović et al., 2022).

### 2.9 Physical and morphological properties of reconstituted proliposomes

The reconstitution capacity of freeze-dried (FD) and spray dried B-NLs and loaded NLs/NCs/NAs-PC was assessed by examining the alterations in their size, polydispersity index (PDI), ZP, and EE following dissolution in distilled water (Sarabandi and Jafari, 2020). Also, morphological and structural properties of reconstituted NLs and NAs were evaluated.

### 2.10 Statistical analysis

The data statistical analysis was performed using the SPSS software (version 19.0, SPSS Inc., Chicago, IL) and one-way analysis of variance (ANOVA). All tests were conducted in 3 replications. Besides, the Duncan's test was employed at 5 % significance level to compare the mean values.

## 3 Results and discussion

### 3.1 Nanovesicles characterisation

#### 3.1.1 Mean particle size, PDI, zeta potential and EE

The most important parameters determining the loading capacity, release, stability, in vivo applications, and bioavailability of loaded food or pharmaceutical compounds are particle size and PDI of the delivery systems (Shah et al., 2020). In this study, the effects of PC loading into NLs and coating the particles with a single layer of CH and different concentrations of alginate secondary coating (A) on size indices, PDI, ZP, and EE were investigated (Table 1). The key results of this section included the following: (i) increase in particle size (from 83 to 92 nm) after PC loading; (ii) increase in particle size after CH (∼144 nm) and alginate coating (216–442 nm); (iii) improvement in the degree of homogeneity (PDI index) after PC loading (∼0.23), CH coating (NCs) and optimal alginate concentration (NAs-0.2 %) from about 0.33 to 0.29; (iv) change in ZP of B-NLs (∼-20 mV) after PC loading (−15 mV) and coating with CH (∼36 mV) and secondary layer (∼-24-38 mV); (v) concentrations above 0.3 % A did not show any effect on the value of ZP index.

**Table 1:** Effects of phycocyanin (PC) loading, chitosan (C) and alginate (A) coating on the mean particle size, PDI, Zeta potential and encapsulation efficiency (EE) of nanoliposomes.

Treatments	Size (nm)	PDI	Zeta (mV)	EE (%)
B-NL	83.1 ± 1.8^f^	0.33 ± 0.02^c^	−20.3 ± 1.1^c^	–
NL-PC_6_	92.3 ± 1.9^f^	0.23 ± 0.01^e^	−15.1 ± 0.4^b^	90.3 ± 3.5^a^
NC-0.4	143.8 ± 5.5^e^	0.29 ± 0.02^d^	35.6 ± 0.6^a^	82.9 ± 2.1^b^
NA-0.1	442.3 ± 24.1^a^	0.71 ± 0.03^a^	−23.7 ± 3.1^d^	73.7 ± 2.1^cd^
NA-0.2	216.1 ± 11.3^d^	0.29 ± 0.02^d^	−33.6 ± 1.5^e^	78.2 ± 1.6^b^
NA-0.3	243.7 ± 8.2^c^	0.34 ± 0.02^c^	−37.3 ± 1.6^f^	77.1 ± 2.4^bc^
NA-0.4	320.8 ± 22.5^b^	0.46 ± 0.03^b^	−38.2 ± 1.4^f^	72.9 ± 2.1^d^

Data are presented as mean ± standard deviation (n = 3) and different letters in the same column indicate significant differences at the 5 % level in Duncan's test. PDI: Polydispersity index; NL-B: Blank nanoliposome; NL-PC: Nanoliposome loaded with phycocyanin; NC: Nanochitosome; NA: Nano-Alosome (NC coated with alginate).

These findings indicate that PC is mainly located within the internal cavity of vesicles, conjugation, deposition, and deposition of polymer layers (C-A) and an increase in membrane thickness, saturation of vesicle surfaces by polysaccharides through electrostatic attraction, and the creation of a dense and compact layer. However, low or high concentrations of A led to an increase in the average particle size and PDI index due to increased particle aggregation and fusion and the formation of some aggregates (W. Liu et al., 2017; Shishir et al., 2019; P. Wang et al., 2025). The EE was evaluated as a measure of the capacity and efficiency of the system to incorporate, protect, and deliver bioactives (Table 1). The value of this index varied in the range of 73–90 %. A decrease in EE (from ∼90 % to 80 %) was observed, as a result of the unintended release of PC during the preparation and coating steps (NCs and NAs) and the occurrence of physical instability in the particles. However, no difference was found between NCs and NAs-0.2. However, higher concentrations of A resulted in the occurrence of physical instabilities, size changes and an increase in PDI, as well as the loss of more PC.

In a similar study, conjugation of CH and pectin (P) onto neohesperidin-loaded NLs resulted in an increase in particle size (from ∼87 to 225 nm), PDI (0.23–0.40), a change in ZP from ∼-24 to ∼20 mV (NCs) and −19 mV (NPs), respectively, and EE (∼64 %) (Shishir et al., 2019). Similar changes (in size, PDI, and ZP) were reported in NLs coated with CH and pectin (P) (Karim et al., 2020), CH and gelatin (GE) (Hosseini et al., 2021), whey protein isolate (WPI) and SA (Ghaleshahi and Rajabzadeh, 2020), as well as CH and SA (W. Liu et al., 2017). However, the encapsulation efficiencies obtained in this study (78–90 %) for NLs, NCs, and NAs were higher or close to the values reported in NLs loaded with oyster protein hydrolysates (Xu et al., 2021), white shrimp peptides (Latorres et al., 2021), ω-3 PUFAs (Hosseini et al., 2021), and camellia seed peptides (CPH) (P. Wang et al., 2025) at about 75, 72, 82, and 83 %, respectively.

According to the results obtained, NCs coated with 0.2 % w/v alginate concentration was selected as the optimal sample for further analyses (along with B.NLs, NLs, and NCs).

#### 3.1.2 Physical, EE and morphological stability

**Fig. 1:**
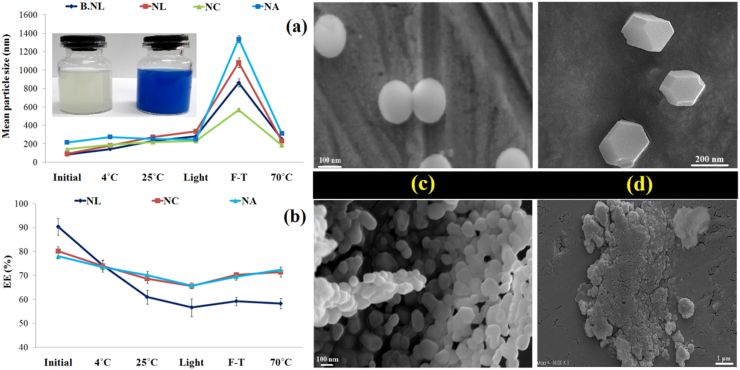
Effects of thermal, light and freeze-thaw (F–T) tensions on the (a) physical and (b) encapsulation efficiency (EE) stability of phycocyanin (PC) loaded nanoparticles. Changes in the morphology of (c) NCs and (d) NAs before and after FT tension.

In this study, the physical stability and EE of NLs and coated samples (NCs and NAs) under different thermal, light, and freezing stresses were investigated (Fig. 1a–d). Overall, several key results were observed: (i) an increase in particle size and a decrease in EE with increasing storage temperature and heating to 70°С; (ii) physical instability and a decrease in EE in samples stored under light exposure (e.g., an increase in particle size in NLs from ∼92 to 335 nm and a decrease in EE from ∼90 to 57 %); (iii) The greatest physical instability and change in particle size after F-T stress (e.g., change in particle size of NLs from about 92 to 1082 nm, and significant decrease in EE (from ∼90 to 59 %) were observed. (iv) F-T stress-induced turbidity, instability, and increase in particle size in B-NLs and NLs are observed in the images; (v) SEM images indicated the presence of spherical nanoparticles with smooth surfaces (Fig. 1c) or relatively polyhedral structures with rougher appearance features (Fig. 1d) for NCs and NAs, respectively. (vi) Monolayer (especially against F-T stress) and bilayer (with alginate) coating significantly improved membrane stability, preserved structural properties, and minimized unwanted release or leakage of loaded PC from the nanocarriers during different stresses. For example, particle sticking, aggregation, agglomeration (Fig. 1c), and sedimentation (Fig. 1c and d) were observed in NCs and NAs samples after F-T stress.

The reasons for the instability of systems during the aforementioned tensions can be summarized as follows: 1) Increased molecular mobility, fluidity, and flexibility of the lipid membrane at higher temperatures leads to reduced physical stability, increased permeability, and leakage of loaded compounds (Akbarbaglu et al., 2024). 2) The accumulation of the hydrated layer on the surface of the phospholipid bilayer as a result of heating can be considered another reason for the increase in particle size (P. Wang et al., 2025). 3) Polymerization of liposomes during storage, especially at high temperatures, and lipid bilayer phase transition (gel to liquid crystal) are also factors that contribute to increasing particle size (Ma et al., 2022). 4) Oxidation of phospholipids as a result of light and chemical changes in lipids is among the reasons for physical instability and reduced EE in samples stored exposed to light (Laein et al., 2024). 5) Mechanical damage and membrane rupture resulting from ice crystal formation during freezing are the main mechanisms of physical instability, increased tendency to agglomerate, particle aggregation and sedimentation, as well as the release of loaded compounds (especially after defrosting) (Akbarbaglu et al., 2024). However, the instability of NAs during F-T stress can probably be attributed to the loosening and loss of strength of the deposited layer on the surfaces of lipid membranes, the reduction of repulsive forces between particles, as well as the formation of networks or aggregates as a result of the binding and attraction of adjacent particles to each other (W. Liu et al., 2017). The most important effects of coating (especially double-layer with A) on improving the strength and stability of nanoparticles include the following: 1) Increased strength and structural rigidity as a result of the physical barrier or layer created by polymers are among the reasons for maintaining the physical stability of nanoparticles and EE during storage under different conditions (P. Wang et al., 2025). 2) The increase in hydrogen bonds as a result of polymer coatings leads to a more compact internal chain of lipid molecules, improved membrane strength, and increased thermal stability. Also, the polymer coating reduces lateral diffusion within the membrane, the rate of compound release and leakage, and susceptibility to oxidation (Haghighi et al., 2018; Karim et al., 2020). 3) Gums and polymers such as CH and alginate act as thickeners in the system and delay the rate of vesicle aggregation and fusion (Cong et al., 2023).

Similar results were observed and reported as a result of the use of membrane stabilizing agents, and the coating of monolayer or bilayer NLs loaded with collagen hydrolysates (Chotphruethipong et al., 2020), oyster protein hydrolysates (Ma et al., 2022), Phloridzin (Haghighi et al., 2018), camellia peptides (P. Wang et al., 2025), and pelargonidin-3-O-glucoside (Shishir et al., 2021).

#### 3.1.3 Biological stability

**Fig. 2:**
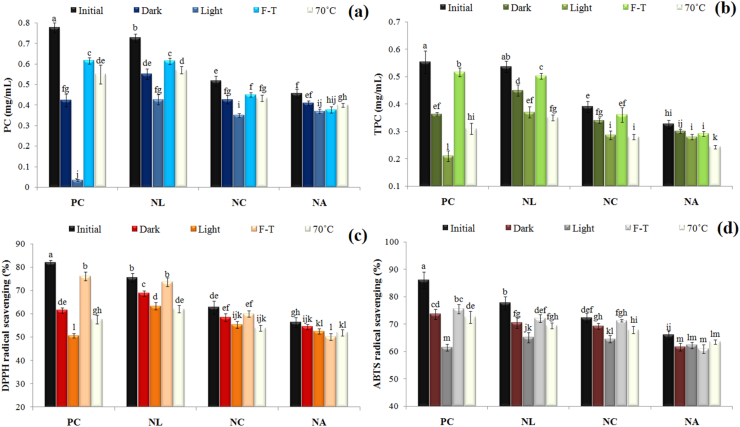
Stability of phycocyanin (a), total phenolic content (c), DPPH (c), and ABTS radical scavenging of free PC-solution and liposomes-loaded (NLs/NCs/NAs-PC) during storage in dark, light, freeze-thaw and 70°С conditions.

Another important and key goal in the design and formulation of lipid nanocarriers is biological stabilization and preservation of bioactives (Shishir et al., 2019). In this study, the amount of bioactives (PC and TPC) and antioxidant activity (AA) (DPPH and ABTS scavenging) and the maintenance of each of these indicators during storage and under different stresses were investigated (Fig. 2a–d). The PC content for NLs, NCs, and NAs varied in the range of 0.46–0.72 mg/mL. First, the encapsulation of PC resulted in its preservation during storage and under different stresses.For example, only about 53 % of PC (in free form) was retained after storage in the dark, whereas loading into NLs (76 %), NCs (86 %), and NAs (92 %) significantly stabilized PC. On the other hand, light had the most destructive effect on PC (more than 95 % degradation) and its decolorization in the free form. The reason can be attributed to conformational changes, decomposition or decolorization of pigments after heat, oxidation or light (Yuan et al., 2022). While loading into NLs, NCs and NAs resulted in the retention of about 58, 69 and 82 % of PC, respectively. The reason can be attributed to the increase in the physical resistance of the membrane, the decrease in the release rate, and the light-absorbing or light-scattering effects of the polymer layers (Shishir et al., 2021). Due to the peptide nature of PC, factors such as conformational changes, partial denaturation, structural and thermal instability are other reasons for its degradation under thermal and freezing stresses (Laein et al., 2024). A similar trend can be observed for phenolic compounds (TPC) (Fig. 2b), where only about 38 % of TPC was retained after storage of the free solution under light exposure, while NLs, NCs and NAs retained more than 69, 75 and 86 % of TPC, respectively. Unlike PC, about 56 % of TPC was retained in the free form after heating at 70°С. Whereas, the use of nanocarriers resulted in the retention of about 65–73 % of phenolic compounds. On the other hand, the least degradation of phenolic compounds occurred under F-T conditions. This indicates the stability of these compounds to freezing conditions. Regarding AA, the amount of these indicators in samples subjected to different stresses was a function of the preservation or destruction of phenolic compounds and PC (Fig. 2c and d). The most damaging effect on AA was related to light. Free solutions exposed to light retained only about 62 % (DPPH) and 71 % (ABTS) of their initial AA. While >85–94 % of AA was retained in NLs, NCs and NAs. Bilayer coating or NAs showed the highest protective effects on bioactives and AA. In addition to the mechanisms described for the physical and structural stability of nanocarriers (3.1.2), factors such as: (i) delay in hydration of the liposomal membrane and increase in membrane stability, (ii) reduction of leakage and unwanted release or retention of EE, and (iii) blocking of oxygen and oxidative reactions in lipids are among the reasons for the preservation of bioactives and AA during various stresses (Cong et al., 2023; W. Liu et al., 2017).

In another study, loading of Pelargonidin-3-O-glucoside into NLs and CH and pectin (P) coated particles was performed. The results showed that the physical stability of the coated bilayer NLs increased towards salt and pH, oxidative, thermal and UV. Also, >96 % of bioactives were retained during storage conditions (Shishir et al., 2021). In another study, NLs coated with CH and pectin (P) and containing neohesperidin (NH) had significantly better physicochemical stability than NL under storage, thermal, pH, ionic, UV, oxidative, and serum conditions (Karim et al., 2020).

#### 3.1.4 *In vitro* release

**Fig. 3:**
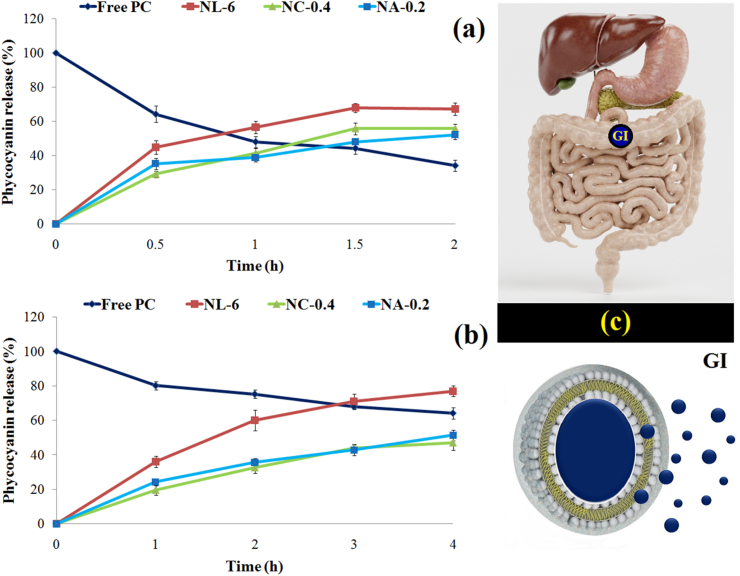
Effects of mono (chitosan) and double (alginate) layer coating on the release of PC loaded inside nanoliposomes during stomach (a) and intestinal (b) digestion. (c) Schematic of the controlled release of PC into the colon.

One of the goals of designing lipid-based systems is to protect loaded bioactives from the damaging conditions of the stomach and improve their targeted, controlled delivery and bioavailability (P. Wang et al., 2025). In this study, the physical strength of the lipid membrane was improved and stabilized by coating with CH/alginate polymers. Finally, the effects of monolayer and secondary coating on the release of loaded PC under gastric (Fig. 3a) and intestinal (Fig. 3b) conditions were investigated. The addition of free PC solution in SGF conditions rapidly led to degradation and a decrease in its detectable concentration from 100 % (initial concentration) to about 60 % (after half an hour of reaction). After 2 h, the amount of PC remaining reached about 34 %. This was despite the fact that at the beginning (the first half hour) a sharp release of loaded PC (∼30–45 %) was observed inside the nanocarriers (especially NLs). However, thereafter, the release rate was gradual and about 67 % (NLs) and 52 % (NAs) of PC were released under SGF conditions, respectively. Protonation and destruction of the lipid membrane can be considered as the reasons for the rapid release of compounds from the NLs structure under gastric conditions (Karim et al., 2020). On the other hand, free PC, due to its peptide nature, showed greater stability in intestinal conditions (SIF). However, coating NLs with CH (NCs) and alginate (NAs) significantly resulted in a slower and more controlled PC release process. PC release from the lipid membrane can be a result of swelling or degradation by bile salts and trypsin. Thus, bile salts can provide binding sites for digestive enzymes through hydrophobic and electrostatic interactions, reducing surface tension, and increasing the fluidity of the liposome membrane (Akbarbaglu et al., 2024). At the same time, trypsin can initiate lipolysis of phospholipids and form unstable micelles, which leads to increased lipid membrane permeability (P. Wang et al., 2025).

The above results indicate an increase in the resistance of the membrane and the physical structure of the nanovesicles to acidic conditions and also to intestinal digestive enzymes after coating with polymers. Generally, CH dissolves in acidic conditions and membrane instability occurs (Gharehbeglou et al., 2024). However, the secondary coating with alginate plays an important role in increasing the structural strength in gastric conditions. However, its higher solubility in alkaline intestinal conditions leads to dissolution and release of the loaded compounds (W. Liu et al., 2017). In another study, researchers reported that CH modification can effectively control the release of peptides during gastrointestinal digestion. As a physical barrier, CH can prevent the interaction between lipid bilayers and digestive enzymes or bile salts. In addition, CH can reduce the permeability and fluidity of the liposome membrane by interacting with the liposome double bond (Pavlović et al., 2022).

In other studies, coating monolayer or bilayer vesicles with biopolymers resulted in the slow or controlled release of loaded compounds such as oyster protein hydrolysates (Xu et al., 2021), salmon protein hydrolysates (Li et al., 2015), neohesperidin (Karim et al., 2020), and vitamin C (W. Liu et al., 2017), and camellia seed peptides (P. Wang et al., 2025).

### 3.2 Proliposomes characterisation

#### 3.2.1 Physicochemical and biological properties

In this study, the effect of liposomal formulation composition (B-NLs, NLs, NCs, and NAs) on PY, physical and functional properties, stability, retention of bioactives, and AA was investigated (Table 2a–c). The PY (48–61 %) was affected by PC loading (decreased) and coating (increased) of nanoparticles. The MC (2.8–3.5 %) and *a*_*w*_ (0.28–0.31) of nanoparticles increased after polymer coating. The use of polymer coating resulted in a decrease in the density (bulk and tapped) of proliposomal particles. Although no difference in HR and CI was observed between the samples, prochitosomes (P-NCs) had a lower repose angle (35°). All samples had high solubility (>91 %), which is an effective factor in particle reproducibility. On the other hand, the average particle size for proliposomes ranged from about 433 nm (B-NLs) to 592 nm (NCs), falling within the nano range (Table 2b). The hygroscopicity was investigated as a key indicator of the stability (physical, oxidative and microbial) of the powders during storage. The value of this index (∼25 %) was higher in coated samples (NCs and NAs) than in B-NLs (about 19 %) (Table 2c). Shear and thermal stresses are effective factors in membrane degradation, structural instability of NLs, increased susceptibility to oxidation (Laein et al., 2024), and loss of biological properties and therapeutic effects of bioactives (especially peptides and peptide-based drugs) (Haque and Adhikari, 2015). The use of polymer coating resulted in increased stability and retention of bioactives and AA. About 93–96 % of the PC, TPC, AA (DPPH and ABTS scavenging) in NCs and NAs were retained during SD (Table 2c). Production efficiency >50 % and MC and a_w_ values obtained can be considered as an indication of acceptable production and suitable microbial stability of the powders, respectively. Also, factors such as particle size, morphological characteristics, and interparticle adhesion or friction are among the factors affecting density and flowability indices (Laein et al., 2024).

**Table 2:** Effects of phycocyanin (PC) loading, chitosan (C) and alginate (A) coating on the physicochemical properties and retention of biological activities in spray-dried nanoliposomes.

(a)	Yield (%)	MC (%)	Aw	BD (g/mL)	TD (g/mL)
B-NL	55.1 ± 2.2^b^	2.8 ± 0.1^c^	0.28 ± 0.01^b^	0.31 ± 0.01^a^	0.41 ± 0.01^a^
NL	48.2 ± 2.4^c^	3.1 ± 0.1^b^	0.29 ± 0.01^ab^	0.30 ± 0.01^a^	0.38 ± 0.01^b^
NC	61.5 ± 2.8^a^	3.5 ± 0.2^a^	0.31 ± 0.02^a^	0.28 ± 0.01^b^	0.37 ± 0.01^b^
NA	56.5 ± 3.2^ab^	3.5 ± 0.1^a^	0.31 ± 0.01^a^	0.27 ± 0.01^b^	0.36 ± 0.02^b^
**(b)**	HR	CI	AR (°)	S (%)	Size (nm)
B-NL	1.30 ± 0.02^a^	0.25 ± 0.01^a^	43.3 ± 1.5^a^	92.9 ± 2.5^a^	433.4 ± 14.5^d^
NL	1.28 ± 0.04^b^	0.22 ± 0.02^b^	40.6 ± 2.3^a^	93.2 ± 1.1^a^	470.5 ± 15.8^c^
NC	1.29 ± 0.02^ab^	0.23 ± 0.01^ab^	35.8 ± 2.6^b^	91.5 ± 2.1^a^	591.3 ± 18.7^a^
NA	1.33 ± 0.02^a^	0.25 ± 0.01^a^	40.3 ± 2.1^a^	92.5 ± 1.4^a^	517.2 ± 17.3^b^
**(c)**	Hg (%)	PC (%)	TPC (%)	DPPH (%)	ABTS (%)
B-NL	19.2 ± 1.4^b^	–	–	–	–
NL	23.4 ± 0.9^a^	86.8 ± 2.3^b^	90.4 ± 1.9^b^	91.8 ± 1.6^b^	91.5 ± 1.7^b^
NC	24.7 ± 1.4^a^	93.7 ± 1.4^a^	94.8 ± 1.8^ab^	96.4 ± 2.1^a^	94.3 ± 1.8^ab^
NA	25.9 ± 1.5^a^	92.8 ± 1.7^a^	92.9 ± 1.2^a^	96.9 ± 1.5^a^	95.1 ± 1.4^a^

Different letters in the same column indicate statistical significant differences (P < 0.05).

MC: Moisture content, Aw: Water activity, BD: Bulk density, TD: Tapped density, HR: Hausner ration, CI: Carr index, AR: Angle of repose, S: Solubility, Hg: Hygroscopicity. NL-B: Blank nanoliposome; NL-PC: Nanoliposome loaded with phycocyanin; NC: Nanochitosome; NA: Nano-Alosome (NC coated with alginate).

In another study, curcumin-containing proliposomes with different ratios of hydroxypropyl β-cyclodextrin (HP-βCD) were produced by SD. The results indicated PY (52–81 %), particle size (100–200 nm), ZP (2–40 mV), and controlled release of curcumin from the proliposomes (Adel et al., 2021). In another study, vitamin D_3_-loaded proliposomes were prepared using the micronized sucrose (MSC) coating technique and a mixture of soy lecithins of varying purity. The phospholipid powders were characterized in terms of *a*_*w*_, MC, solubility, hygrometry, moisture absorption isotherms, and the amount of residual VD_3_. The results indicated that the powders had high MC, *a*_*w*_, and solubility (Chaves and Pinho, 2020). In another study, NLs (uncoated or chitosomes) containing black carrot anthocyanins were produced by SD. The PY (34–52 %), particle size (11–19 μm), MC (3.8–5 %), and *a*_*w*_(0.11–0.19) were obtained under the influence of the study variables (Guldiken et al., 2019).

#### 3.2.2 Color indices

**Fig. 4:**
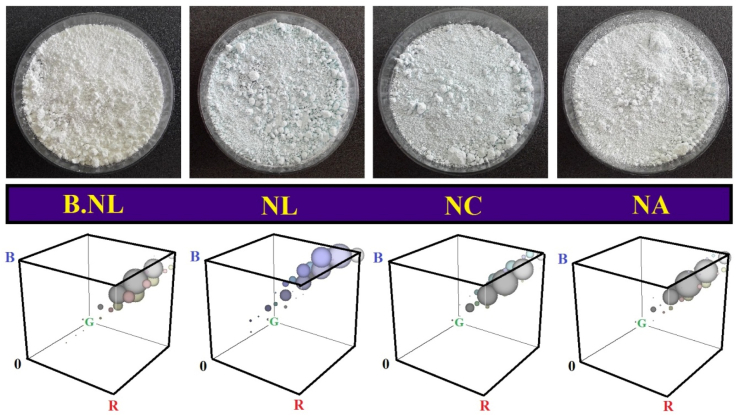
Appearance characteristics and color histogram of spray-dried empty (a), NLs-PC (b), NCs-PC (c), and NAs-PC (d).

Preservation of color parameters is another goal of encapsulation of bioactives and effective on the qualitative characteristics of formulations (Laein et al., 2024). In this study, the appearance properties, parameters and color histogram of the powders (B-NLs/NLs/NCs/NAs) were evaluated (Table 3, Fig. 4). The lightness index (L value) of the coated samples was higher than that of SD-BNLs. No significant differences were observed in the a-index (Red = +, green = -). However, the b-index tended towards negative (blue) values in PC-containing proliposomes. This finding indicates the presence and effects of the intrinsic color of PC on the color parameters of the particles. However, the chroma index was reduced in the samples obtained from the coated particles. The color histogram of the particles also indicated the dispersion of blue pigments, especially in the proliposomes obtained from the NLs (without coating). The incorporation and coating of PC within the particles (coated with biopolymers) can also be effective in protecting them from thermal/dehydration stresses during SD.

**Table 3:** Color properties of spray-dried nanoliposomes loaded with phycocyanin.

Treatment	L∗	a∗	b∗	Hue angle (°)	Chroma
B-NL	75.6 ± 1.21^c^	−0.36 ± 0.03^a^	3.32 ± 0.17^a^	96.2 ± 0.71^c^	3.32 ± 0.18^a^
NL	76.3 ± 0.91^bc^	−1.48 ± 0.06^b^	−0.14 ± 0.02^c^	185.2 ± 2.63^a^	1.49 ± 0.07^b^
NC	78.1 ± 0.94^b^	−1.62 ± 0.02^c^	−0.04 ± 0.01^c^	181.4 ± 1.96^a^	1.62 ± 0.02^b^
NA	80.5 ± 0.72^a^	−0.42 ± 0.02^a^	1.04 ± 0.08^b^	112.5 ± 3.21^b^	1.13 ± 0.07^c^

Data are presented as mean ± standard deviation (n = 3) and values denoted by different letters within each column are significantly different (p < 0.05). NL-B: Blank nanoliposome; NL-PC: Nanoliposome loaded with phycocyanin; NC: Nanochitosome; NA: Nano-Alosome (NC coated with alginate).

#### 3.2.3 Thermal properties

**Fig. 5:**
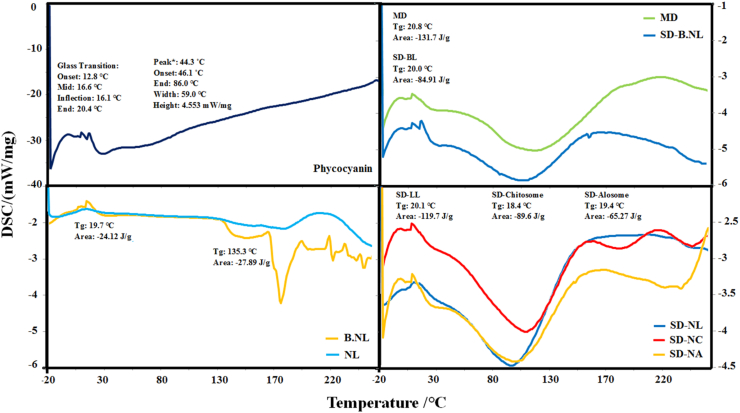
Thermal properties (DSC) of pure phycocyanin (PC), blank (B-NL), loaded (NLs) and spray-dried samples (NLs/NCs/NAs-PC).

In this study, MD was used as a matrix during SD of control (B-NLs) and PC-containing liposomes (NLs/NCs/NAs) (Fig. 5). The thermograms of all of them had a broad endothermic peak, related to moisture evaporation (Pirayavaraporn et al., 2012; S. Wang et al., 2017). This peak for MD was observed at a higher temperature (114.6°С) than SD-B-NLs (103.6°С), SD-NLs (93°С), SD-NCs (104.5°С), and SD-NAs (95.5°С). The presence of a single peak in the proliposomes indicates compatibility between them and MD and the formation of a compact and homogeneous structure without any phase separation, in which the liposomes are located in a dominant MD matrix. Also, the decrease in T_g_ of the peaks in them compared to MD is due to their reduced structural order. The higher T_g_ in SD-NCs and SD-NAs compared to SD-B-NLs is probably because the coating of liposomes with biopolymers reduces their structural disorder. Ang et al. (2024) also reported that the MD endothermic peak in spray-dried SD-NCs (containing apigenin) powder shifts to lower temperatures (Ang et al., 2024). In another study, fish peptides were loaded into NCs. DSC findings indicated increased electrostatic interactions between CH and lecithin molecules, leading to an increase in the melting (T_m_) and decomposition (T_d_) temperatures of the peptide-liposome hybrid system (Ramezanzade et al., 2021). Also, in another study, *Lactobacillus rhamnosus*was loaded into NLs coated with CH-gelatin (GE) polyelectrolytes. According to the DSC results, the deposition of CH-GE polyelectrolytes on the surface of NLs changed the physical properties of the phospholipid bilayer. Also, the increase in T_m_ from 120 to 127.5°С in CH-GE-coated NLs with *L. rhamnosus* made this system more stable than uncoated liposomes (Hosseini et al., 2022).

#### 3.2.4 Structure analysis (FTIR)

**Fig. 6:**
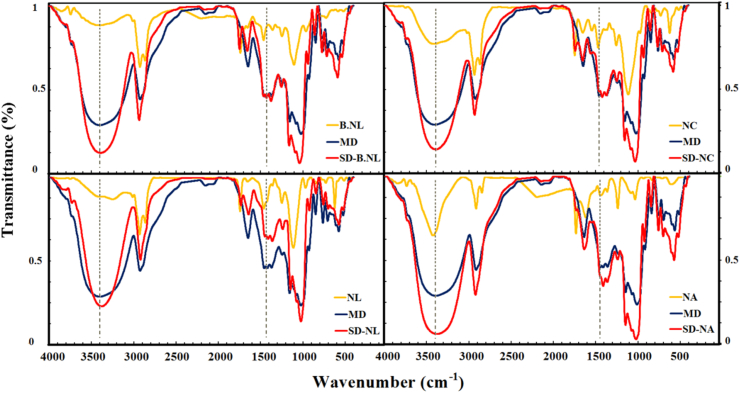
Effects of mono (chitosan) and double (alginate) layer coating on the chemical (FTIR) structure of nanoliposomes (NLs/NCs/NAs) loaded with PC.

The structural features of the nanoparticles were investigated to determine the effects of PC loading into the vesicles, coating with polymers (CH/alginate), and SD within the MD matrix (Fig. 6). The principal functional groups identified in PC, as reported by Zheng et al. (2020), include the following: a peak at 3305 cm^−1^ corresponding to amide A (N-H stretching), 2931 cm^−1^ representing amide B (C-H and O-H stretching), and the amide I region ranging from 1700 to 1600 cm^−1^, notably characterized by the α-helix structure at 1650 cm^−1^. Additional significant absorption bands include the -NH_2_ bending vibration at 1543 cm^−1^, as well as -C-O stretching and -OH deformation observed at 1049 cm^−1^.

The chemical structure of the empty NLs (B-NLs) showed several main peaks. The most important peaks are at frequencies of 3395 cm^−1^ (O-H stretch), 2929 cm^−1^ (symmetrical or asymmetrical stretch vibrations of CH_2_), 1735 and 1649 cm^−1^ (stretching vibrations of the C=O bond of the aliphatic ester in the region connecting the hydrocarbon chain and the head group), 1460 cm^−1^ (vibrations of the CH_2_ group), 1354 cm^−1^ (C-H stretching vibration), 1243 and 1099 cm^−1^ (symmetrical stretching vibrations of PO^2−^ in phospholipids), and finally, the frequency of 1959 cm^−1^ (asymmetric stretching vibrations of the choline group (N ^+^ CH_3_) in the polar part of phosphatidylcholine)(Karim et al., 2020; Shishir et al., 2021).

Overall, PC loading in NLs resulted in the following changes (Chaves and Pinho, 2020; Gómez-Mascaraque et al., 2017; Shishir et al., 2021): Shift and increase in intensity of peaks 1) from 3395 to 3246 cm^−1^ (due to hydrogen bonds between O-H and N-H groups); 2) from 2929 to 2925 cm^−1^ (ionic reactions between phosphatidylcholine (NLs) and peptides (PC) and placement of PC in the space of the monolayer membrane (NLs)); 3) from 1735 to 1649 to 1738 and 1649 cm^−1^ (hydrogen bonds between PC and the carbonyl group of phosphatidylcholine (NLs); 4) from 1243 to 1099 cm^−1^ (B-NLs) to 1244 and 1108 cm^−1^ (NLs) (hydrogen bonds with PO_2_ and lipid polar regions); 5) from 1959 cm^−1^ (B-NLs) to 1963 cm^−1^ (NLs) (PC placement within the inner cavity of NLs and polar parts of phosphatidylcholine).

The most important groups related to CH can be observed at frequencies 3442 cm^−1^ (O-H stretching vibrations), and 1651 and 1601 cm^−1^ (vibration of C=O of the amide group and protonated amine) (Bai et al., 2011). Coating NLs with CH resulted in the shift of some peaks. The peaks at frequencies 3324, 2925, 1738, 1649, 1459 and 1244 cm^−1^ were shifted to 3399, 2924, 1737, 1643, 1460 and 1247 cm^−1^, respectively, and the intensity of the peaks (in the second region) increased. These changes indicate: 1) the placement of the CH layer on the outer surface of the NLs membrane; 2) an increase in hydrogen bonds between the carbonyl regions of the monolayer and CH; 3) a more compact and denser structure; and 4) a decrease in the fluidity of the acyl chain in the monolayer membrane (X. Liu et al., 2024; Shishir et al., 2021). In alginate, salient spectral features comprise a broad absorption band at 3440 cm^−1^, attributed to hydrogen bonding involving N-H stretching and bending of C-H groups, along with distinct peaks at 1618 cm^−1^ (C=O stretching), 1419 cm^−1^ (N–H and C–N stretching), and 1030 cm^−1^. The absorption peak at 1030 cm^−1^ may indicate the presence of amide functionalities or suggest potential perturbations in the NH^3+^ group of lysine residues incorporated within the protein structure (Zolqadri et al., 2025).

Secondary coating resulted in the following changes in FTIR spectroscopy: increased peak intensity and frequency shifts from 3399, 2924, 1737, 1643, 1460 and 1247 cm^−1^ (NCs) to 3433, 2925, 1795, 1625, 1453 and 1249 cm^−1^ (NAs), respectively. Alginate is an anionic polysaccharide due to the presence of guluronic and mannuronic acid residues. The alginate coating of NLs relies primarily on electrostatic interactions and hydrogen bonding. The electrostatic interaction occurs between the negatively charged alginate and the surfaces of NCs. Also, the conjugation of the alginate functional groups with the amine (-NH^+3^) of CH takes place (Karim et al., 2020; Shishir et al., 2019). For MD, serving as the base matrix, key structural regions were identified as follows: O-H stretching at 3395 cm^−1^, C-H stretching at 2926 cm^−1^, C=O stretching at 1648 cm^−1^, CH_2_ bending at 1457 cm^−1^, O-H bending at 1371 cm^−1^, C-O stretching and C-O-H bending at 1082 and 1022 cm^−1^ respectively, along with skeletal vibrations of the pyranoid ring observed at 707 and 576 cm^−1^ (Akbarbaglu et al., 2024).

SD and conversion of nanoparticles (B-NLs/NLs/NCs/NAs) into proliposomes led to the following changes: 1) An increase in peak intensity in the first regions (2900-3100 cm^−1^ region) as well as a shift and increase in peak intensity in the frequency ranges of 1460-1457 cm^−1^ and 1249-1243 cm^−1^ were observed. 2) The overall spectrum and FTIR pattern of all spray-dried samples are a function of the structural features of pure MD. This finding indicates the location and distribution of lipid nanoparticles within the MD matrix (Ma et al., 2022). The reasons and mechanisms associated with these changes can be attributed to the formation of hydrogen bonds, hydrophobic and electrostatic interactions between nanoparticles (especially NLs/NCs) and MD (Akbarbaglu et al., 2024). In similar studies, structural changes, lipid membrane compaction, and the formation of some hydrogen and hydrophobic interactions were reported as a result of the production of proliposomes containing oyster protein hydrolysates (Ma et al., 2022), vitamin D_3_ (Chaves and Pinho, 2020), canthaxanthin (Ravaghi et al., 2017), and food waste compounds (collagen hydrolysate, pomegranate peel, and shrimp lipid extract (Marín et al., 2018).

#### 3.2.5 Morphological properties

**Fig. 7:**
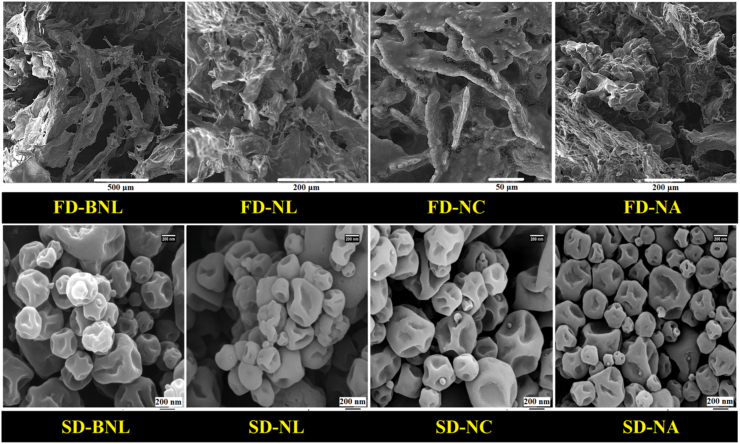
Morphological properties of freeze-dried (FD) and spray-dried (SD) blank (empty) and nanoliposomes (NLs/NCs/NAs) loaded with PC.

In this study, to better investigate the effects of coating NLs with biopolymers (without MD matrix), the morphological characteristics of freeze-dried particles were investigated (Fig. 7). Also, SEM analysis was performed on SD particles. The images of freeze-dried B-NLs indicated the production of thin, lamellar, porous, and brittle structures. This was while adding PC and loading it inside NLs led to changes in the morphology of the particles. On the other hand, the coated samples (NCs and NAs) showed thicker, rougher, denser and stronger structures. These results indicated the effects of using polymers on increasing the strength and morphological changes in NLs. On the other hand, the SD samples showed nanoparticles of relatively different sizes, wrinkled with surface indentations. Also, the obtained images are in confirmation of the results of particle size (Table 2b). The reasons for the production of wrinkled particles include the rapid evaporation of moisture during drying, and the rapid formation of a skin or film around them (Ma et al., 2022). The presence of wrinkled proliposomal particles indicates their physical and structural stability during SD process (Guldiken et al., 2019). In another study, Carboxymethyl chitosan-coated proliposomes (CMCS) were produced for stabilization and targeted delivery of coix seed oil. The results showed that the coated proliposomes had more homogeneous particles, smoother surfaces, and better stability compared to other samples (Bai et al., 2011).

#### 3.2.6 Physical and morphological properties of reconstituted proliposomes

**Fig. 8:**
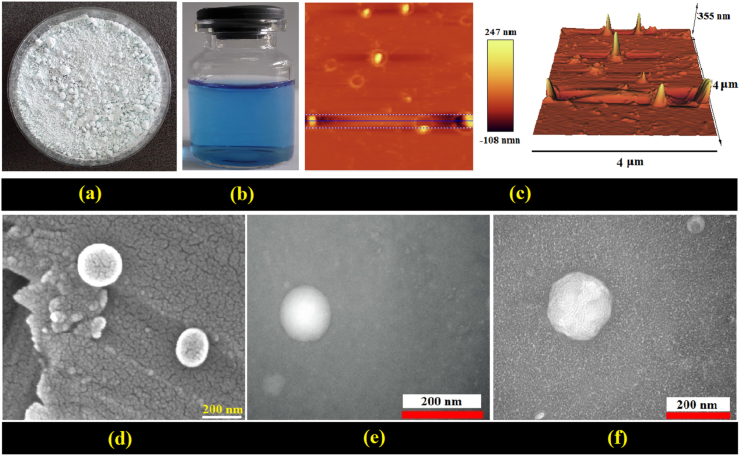
Appearance of (a) powdered, liquid form (b), AFM (c), SEM (d), TEM (e) of reconstituted spray-dried (SD) NLs-PC. (f) TEM image of reconstituted spray-dried (SD) NAs-PC.

In this study, the effects of nanovesicle coating on the retention of physical properties and EE after reconstitution were investigated. Also, the effects of each SD and FD processes on the above properties were compared (Table 4). First, the changes in particle size after reconstitution of FD samples were greater than SD. This finding indicates the greater instability of the brittle and porous structures of FD samples and the occurrence of sticking, agglomeration and particle coalescence phenomena after reconstitution (Akbarbaglu et al., 2024). On the other hand, the particle size in the reconstituted NCs changed to a lesser extent compared to the other samples (Table 4a). Factors such as the physical stability of the membrane, its stiffness and strength, and the initial particle size can be considered as reasons for this finding (Ma et al., 2022). Similar results were observed for PDI, in that R-SD (NCs) samples had more homogeneous structures compared to other samples. ZP changes were also lower for R-SD samples than for R-FD. The EE index of FD-NLs decreased significantly after reconstitution (Table 4b). These changes indicate the presence of a fragile and unstable membrane, as well as the loss of a significant portion of the loaded PC after reconstitution of the particles. Meanwhile, the EE changes in NCs and NAs samples were clearly reduced. This also indicates the effects of polymer coatings on maintaining membrane stability during drying stresses (FD and SD) and preventing unwanted leakage or leakage of compounds during regeneration (Laein et al., 2024). The production of clear and stable NLs (without turbidity and sediment) after the reconstitution of SD particles can be observed (Fig. 8b).

**Table 4:** Effects of chitosan (C) and alginate (A) coating on the physical stability and EE-retention of phycocyanin (PC) loaded FD and SD-nanoliposomes after reconstitution.

(a)	Initial	Z-average (nm)		Initial	PDI	
		R-FD	R-SD		R-FD	R-SD
B-NL	83.1 ± 1.8^cC^	168.2 ± 8.5^bA^	139.1 ± 6.5^cB^	0.338 ± 0.02^aA^	0.347 ± 0.02^bA^	0.329 ± 0.01^bA^
NL	92.4 ± 1.9^cC^	181.7 ± 3.8^bA^	150.3 ± 3.5^bcB^	0.225 ± 0.01^cB^	0.334 ± 0.01^bA^	0.324 ± 0.01^bA^
NC	143.8 ± 5.4^bC^	182.8 ± 6.1^bA^	161.2 ± 5.7^bB^	0.298 ± 0.02^bA^	0.325 ± 0.01^bA^	0.307 ± 0.01^bA^
NA	216.1 ± 11.3^aC^	398.5 ± 16.2^aA^	295.7 ± 15.1^aB^	0.295 ± 0.02^bC^	0.384 ± 0.01^aB^	0.418 ± 0.02^aA^
**(b)**	Initial	**Zeta-P (mV)**		Initial	**EE (%)**	
		R-FD	R-SD		R-FD	R-SD
B-NL	−19.9 ± 1.2^cA^	−19.7 ± 0.6^cA^	−21.3 ± 1.7^cB^	–	–	–
NL	−15.1 ± 0.4^bB^	−12.9 ± 1.4^bA^	−17.3 ± 0.9^bC^	90.3 ± 3.5^aA^	69.9 ± 1.4^bC^	82.3 ± 1.2^aB^
NC	35.6 ± 0.7^aA^	34.8 ± 0.2^aA^	36.1 ± 0.8^aA^	80.2 ± 1.8^bA^	74.1 ± 0.9^aC^	76.8 ± 1.2^bB^
NA	−33.7 ± 1.5^dA^	−31.4 ± 2.1^dA^	−30.9 ± 0.7^dA^	78.1 ± 1.2^bA^	71.4 ± 1.1^bC^	74.6 ± 1.5^cB^

Data are presented as mean ± standard deviation (n = 3) and values denoted by different letters within each column (lowercase letters) and raw (uppercase letters) are significantly different (p < 0.05). EE: encapsulation efficiency; B: Blank; R: Reconstituted; FD: Freeze-dried; SD: Spray-dried.

On the other hand, the topographic (AFM), morphological (SEM), and structural (TEM) characterizations of the reconstituted particles indicate the preservation of qualitative characteristics, stability (production of discrete particles), and size in the reconstituted samples (confirming the results in Table 4) (Fig. 8c–f). Some of the factors that make proliposomes unstable after reconstitution are as follows: (i) most NLs do not have the same particle size before and after reconstitution and may be affected by FD/dehydration stresses. (ii) There is a greater release of hydrophilic drug compounds (from the central space of the vesicles) than lipophilic ones (trapped and bound within the unilamellar membrane). This can affect the EE of the particles after reconstitution (Shah et al., 2020). The stability of coated particles can be attributed to the formation of more hydrogen bonds (by polymers), greater compaction of the lipid internal chains, increased mechanical, thermal, and oxidative resistance, and reduced lateral diffusion within the membranes (Cong et al., 2023).

In another study, the effects of coating NLs with alginate on the preservation of structural properties and reconstitution ability during drying (FD/SD) were investigated. The results indicated the protective effects of the alginate biopolymer on the physical (size) and morphological stability of the particles after reconstitution (Gómez-Estaca et al., 2021). In the study by Sepúlveda et al. (2021), the effects of adding trehalose to liposomes containing soy lecithin loaded with tilapia protein hydrolysates dried by SD were investigated. The particle size increased from 215 to 250 nm in the initial liposomes to 258–314 nm after SD depending on the trehalose concentration. Also, the spray-dried liposomes loaded with hydrolysates and stabilized with trehalose maintained their membrane stability and spherical shape after 11 months of storage at 4 °C (Sepúlveda et al., 2021).

## 4 Conclusions

In the present study, the effects of coating (monolayer and bilayer) of PC-loaded NLs with CH and alginate polymers on physical properties and EE retention (during thermal, light and freezing stresses), structural, stability (bioactives and biological activity), morphology, and release (under digestive conditions) were investigated. Also, chemical (FTIR), thermal (DSC), and morphological (SEM) properties of the produced proliposomes before and after reconstitution were analyzed. The physical properties and stability of NLs were affected by the alginate concentration. On the other hand, the bilayer coating significantly increased the stability and strength of lipid membranes, resistance to thermal, light, and freezing stresses, and controlled release of PC during gastrointestinal digestion (stomach and intestines). The properties of proliposomes (PY, physical stability, solubility, flowability, particle size, retention of bioactives (PC and TPC) and AA (DPPH and ABTS scavenging), histogram and color parameters were affected by liposomal formulation. Thermal (DSC) and chemical (FTIR) characterizations indicated the effect of PC loading inside the nanoparticles, and confirmed the coating and complexation of polymers on the lipid membrane surface. Morphological characterizations of the particles (freeze-dried) indicated a change in the structural characteristics of the particles (FD) from thin and brittle sheets (B-NLs) to strong, thick and rough structures (especially in NAs). While the SD samples showed relatively different sized, wrinkled particles, the polymer coating significantly preserved the physical, EE, and morphological (SEM, AFM, and TEM) properties of NLs after reconstitution. The results of this study can be used in the design, formulation, and production of proliposomes (stable with controlled release) containing natural pigments, hydrophilic nutrient compounds, and peptide-based drugs. However, further studies are still necessary regarding the use of other membrane protective compounds (types of stabilizers, cryoprotectants, and thermoprotectants), nutritional and in-vivo evaluations.

## CRediT authorship contribution statement

**Zahra Akbarbaglu:** Conceptualization, Methodology, Investigation, Writing-original draft. **Fardin Tamjidi:** Conceptualization, Methodology. **Khashayar Sarabandi:** Supervision, Data curation, Review & editing. **Seid Mahdi Jafari:** Supervision, Project administration, Resources, Review & editing.

## Declaration of competing interest

None.

## Data Availability

Data will be made available on request.
